# Evaluation of Realistic Sensation Using Biological Reaction Measurements for Food Videos Presented by KMMD

**DOI:** 10.3390/s21227670

**Published:** 2021-11-18

**Authors:** Keisuke Tomono, Yutaka Ishibashi, Akira Tomono

**Affiliations:** 1 Department of Information Media Technology, Tokai University, 2-3-23 Takanawa, Minato-ku, Tokyo 108-8619, Japan; keisuke.tomono0829@gmail.com; 2 Graduate School of Engineering, Nagoya Institute of Technology, Nagoya 466-8555, Japan; ishibasi@nitech.ac.jp

**Keywords:** food image, smell, saliva, pupil, electrocardiograph, NIRS, Oxy-Hb

## Abstract

We have prototyped a KANSEI multimedia display (KMMD) that is able to release scent through the screen in order to realize collaboration between images and scents. Two types of “sukiyaki” food videos were presented to subjects using this device, and a method for objectively evaluating the realistic sensation of the food videos was examined using biological reaction measurements. The sukiyaki scent was added to one type of video to improve appetite. Viewers’ saliva flow rate, line of sight, pupil diameter, autonomic nerve activity, and cerebral blood flow were measured at the same time, and changes in these measured values were analyzed. As a result, the scent was effective in improving the sensation, as if the food was present in front of the eyes and increasing the saliva flow rate. Additionally, in a realistic scene, it was found that the line of sight follows the performer’s eating behavior as if the viewers themselves are eating. The sympathetic nervous system temporarily increases, mydriasis occurs, and the frontal lobe is activated. Furthermore, the possibility of objective evaluation of realistic sensations was demonstrated by the correlation between appetite, accompanied by salivary sensation, and the biological reaction measurement results.

## 1. Introduction

As eating is a basic behavior, humans have a strong desire for delicious food. This is why the food industry is expected to grow more and more steadily. Suppliers of foodstuffs try to develop and use various video advertisements that make food look more delicious. For example, cooking and eating shows have always enjoyed high popularity in the TV industry. On the other hand, recently, with the development of technology for displaying scents, the popularity of food advertisements using scents is gradually increasing [1]. For example, by showing videos with the scent of curry powder in food stores, we can expect an increase in sales of the related foodstuff. In addition, as cooking videos are effective in increasing our appetite, they are expected to urge those people with little appetite due to aging or anorexia to eat regularly. In this way, food video contents that affect human senses could have great potential in terms of multiple uses in the food industry. However, there are tasks yet to be considered regarding the development of displays to make dishes look more realistic, their contents, and the method of evaluation to measure realistic sensations.

In order to solve these problems, some academic societies have been working on these issues. For example, the Institute of Electrical Engineers of Japan established a research and development committee on olfactory systems, olfactory displays, and KANSEI multimedia in 2005 as a forum for exchanging opinions between sensor and VR researchers [2]. This result was summarized by Nakamoto et al. and published as *Human Smell Interface: Smell Sensing and Presentation* [3]. Research on the psychological effects of scented images and techniques for presenting scents from a distance using an air cannon are also introduced in this book. The Virtual Reality Society of Japan established the Aroma, Taste, and Biological Information Research Committee in 2008 to promote research activities related to the application of scents in VR [4]. In Europe, the DOS (Digital Olfaction Society), which studies the basics and applications of the sense of smell, including olfactory displays, has been active since 2013 [5]. Recently, as a method of emitting a scent from near the image object, a combination of HMD and an olfactory display has often been used [6].

We have proposed and prototyped a device called KMMD (Kansei multimedia display) that enables the discharge of scents from the screen for the purpose of realizing collaboration between images and scents on large-screen displays such as electronic advertisements [7]. We are also launching the experiment to verify how effective showing multisensory information is in improving the impression of “being there” with the products [8].

In this study, food videos of the popular Japanese dish “Sukiyaki [9]” were presented using the KMMD, and then the scent of food was presented alongside a scene where we are apt to associate the food and the scent. It is usual to conduct a subjective evaluation study using a questionnaire when investigating how effective food videos with scents are in terms of the impression of “being there.” However, there are several difficulties associated with this method. For example, there is a time lag before subjects of the questionnaire give the answer, as they have to focus on the videos while contents are shown to them. Therefore, real-time evaluation is difficult to achieve. For this reason, in this study, we conducted a biological reaction measurement while subjects watched content, in addition to giving them a questionnaire to ask about their impression of “being there” with food videos. We also considered the possibility of objective evaluation based on this measurement. If this biological reaction measurement is applicable for evaluating the impression of being there, we are able to reflect the result to the media content, which will bring about more effective electronic advertisements.

We often experience that saliva is produced when we are hungry or when we see our favorite food. This is a vital reaction induced by the process whereby visual stimulation from food images elicits recollection of past episodes relating to eating [10]. Therefore, we assumed that the impression of “being there” is enhanced when cross-modal phenomena, such as sense of taste and saliva release caused by visual and olfactory stimulation, are observed. In this sense, we conducted an evaluation of “being there” based on measurement of biological reactions relating to saliva release. The equipment for biological reaction measurement is as follows: electronic balance for measuring saliva flow rate; line-of-sight measurement equipment to measure both gaze point and pupil diameter; electrocardiograph to measure the autonomic nervous activity; and multi-channel NIRS to measure cerebral blood-flow change. This equipment was used in parallel for one subject for data collection. In analyzing and comparing these measurement results, we also considered a simpler biological reaction measurement that is useful for evaluating realistic sensations.

The remainder of this paper is organized as follows. Section 2 explains the experimental environment, measuring instruments, and the videos used for evaluation. Section 3 describes the experimental method. Section 4 explains the experimental results and considers the possibility of objective evaluation of realistic sensations, and Section 5 provides a summary and discusses and future issues.

## 2. Environment and Equipment for Experiment

We need to consider the following two elements that produce an impression of being there [11]: the physical information of the outside world (external element), which is sensed via sensory organs, and the sensory element (internal element), which is produced inside the brain according to the memory of senses accumulated there based on past experience or learning. For the enhancement of the impression of being there, it is necessary to make the external element as realistic as possible. This leads to the formation of the internal element, enabling the brain to sense a virtual world. In this study, we used audiovisual information as well as olfactory information in the environment with little additional stimulation from the outside world, as we targeted for evaluation food videos that enhance eating images.

### 2.1. Kansei Multimedia Display Device (KMMD)

**Figure 1:**
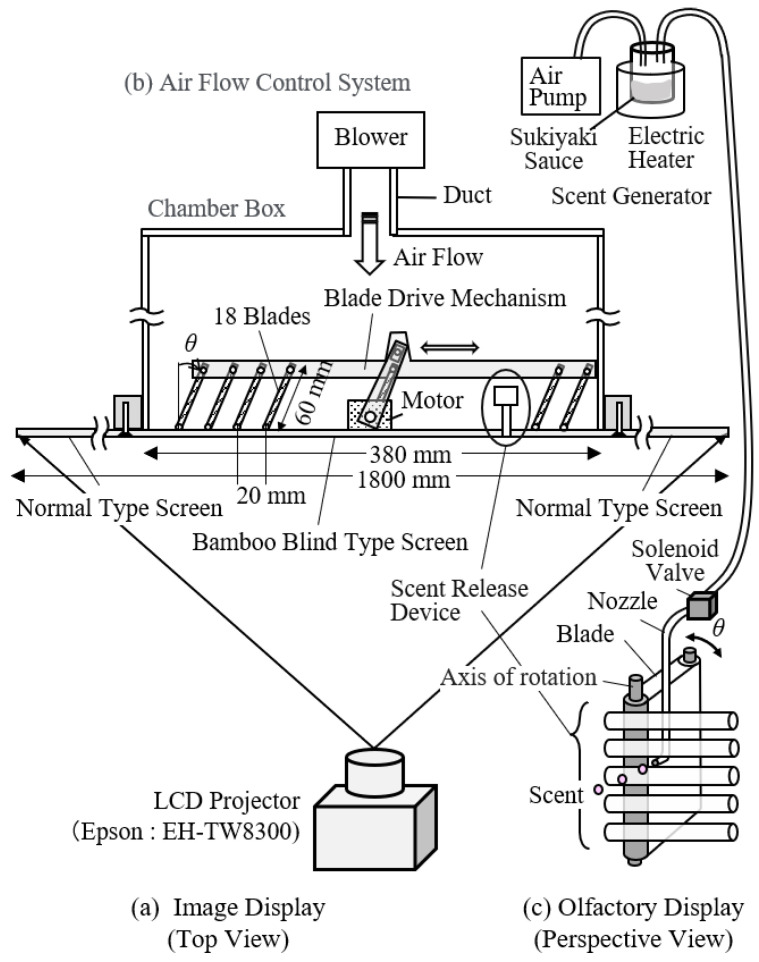
Device configuration of KMMD using a bamboo blind-type screen.

KMMD was designed with the basic concept of emitting a scent through the screen from the vicinity of the image object reminiscent of the scent on the screen. Therefore, a psychological effect is expected as if there is a food with a good scent there [12]. Figure 1 shows the KMMD used in the experiment. The screen is made of a special board with a size of 180 cm × 120 cm, with a bamboo shade-like screen (38 cm × 18 cm) in the center. The scent comes out of a slit of bamboo blind. The picture is projected by an LCD projector (Epson: EH-TW8300). The projector has a resolution of 1920 × 1080 pixels (corresponding to Full HD) and a maximum luminance of 2500 lm.

In the rear of the abovementioned bamboo blind-like screen, an airflow control device is installed. The device consists of a chamber box, multiple blade-plate rotation mechanisms installed in the box, a nozzle to discharge scents, a blower that increases air pressure, and a duct. The blade plate is driven by a motor. By controlling its direction, it is possible to release the air flow in an arbitrary direction from the rear side of the screen. A scent-release system and an air pump are connected to the scent-generation nozzle so as to supply scent in coordination with videos. The scent presentation method will be described in detail in 2.5.

### 2.2. Evaluation Laboratory

**Figure 2:**
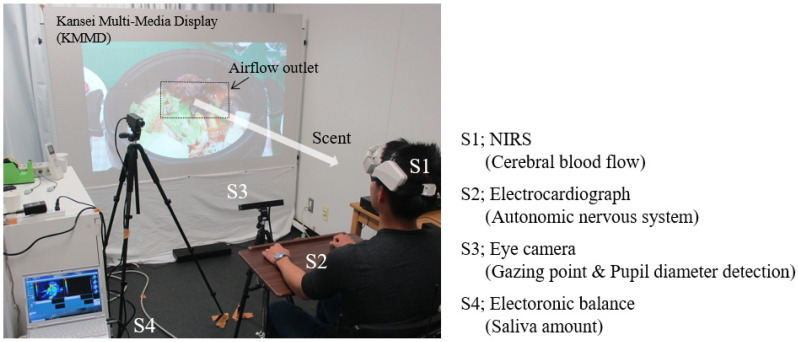
Experimental environment with KMMD and measuring devices.

Figure 2 shows a psychological experiment evaluation room in which the KMMD and the measuring device were set up. To display visual, auditory, and olfactory stimulation, a quiet and calm environment separated by white curtains with an area of 10 m^2^ was used. During the experiment, the laboratory was kept at a temperature of 24 °C and a humidity of 50% so that subjects would feel comfortable [13]. Since various measuring devices were attached on the subjects, the psychological effects of the environment are described in 3.0.

### 2.3. Method of Measurement for Biological Reaction

#### 2.3.1. Measurement of Saliva Flow Rate

Saliva mainly comes from salivary glands. Salivary glands are categorized into parotid gland, submandibular gland, and sublingual gland based on their location [14]. There are other minor salivary glands located in the oral mucosa or the tongue. Secretion capacity of saliva is related to autonomic nerves. When the parasympathetic nerve is dominant, the serous saliva flow rate increases. When the sympathetic nerve is dominant, a small amount of mucous saliva is secreted [15]. Secretion of saliva occurs reflectively. There are two cases: when food gets into the oral cavity, saliva comes out due to the mechanical stimulation or stimulation of flavor; when we see or smell food, we are apt to associate it with appetite, which leads to saliva secretion [16].

Based on this, we came to think that if saliva release occurs after we see an image of food and are reminded of appetite, this phenomenon means that there is a high sense of the impression of “being there”. For this reason, we conducted a measurement of saliva secretion.

The method for measuring saliva release directly is as follows [17]. Spit out saliva in a container for a certain period of time and to measure saliva secretion. In this experiment, we adopted the Watts method [18], which is a way to measure absorbed saliva by inserting absorbent cotton onto the lower tongue, in a modified way, as measurement had to be conducted while keeping subjects focused on the video. Concretely, we used a saliva-absorption sheet with a rough size of two mm thick and four cm^2^ area (Salivatol by OJI KINOCLOTH Corporation, Tokyo, Japan). This sheet is made of cellulose mixed with polyester polyethylene composite fiber. The sheet has excellent saliva absorption and water-retention capacity, as well as good stability in the oral cavity. It causes little discomfort, such as stickiness. The difference between the weight of the sheets before and after the display time was measured using a high-precision weight scale (a sonic-type analytical scale by Shinko Denshi Corporation: HTR-220). Minimum weighing capacity is 0.0001 g. Details of the saliva flow-rate measurement procedure will be described in Section 3.

#### 2.3.2. Measurement of Line of Sight and Pupil Diameter

Humans have a propensity to turn their eyes to an object they are interested in. Therefore, measurement of line of sight is effective in presuming what subjects are watching. The pupil is a hole surrounded by the iris of the eyes. The iris dilator muscle is a smooth muscle located radially in the iris of the eyes. When the muscle shrinks due to sympathetic hypertonia, the pupil becomes dilated. On the other hand, the smooth muscle located on the circumference of the iris of the eyes is called the iris sphincter. When it shrinks due to parasympathetic hyperactivity, the pupil contracts [19]. The pupil has a propensity to change its size depending on the light strength (pupillary light reflex), while miosis occurs when we see nearby things (near reflex). Moreover, mydriasis occurs due to mental excitement or strain [20]. Based on this, it can be assumed that a measurement of the change of pupil diameter while watching videos will be useful for presuming the mental state of the viewer.

We used the eye mark recorder EMR ACTUS [21] by nac Image Technology Corporation (Figure 2—S3) as the non-wearable line-of-sight measuring device (eye camera). Based on image-measurement technology, it is convenient for order to making viewers focus on the video. The device enabled us to measure a binocular gaze point and pupil diameter at the same time. The detection rate is 60 Hz for both eyes. The detection range is 50–80 cm from the device. The eye-movement-detection accuracy is 0.5 deg, and the resolution is 0.3 deg. The pupil diameter resolution is 0.1 mm. The measurement data were processed by the analysis software EMR d-Stream. The point where the line of sight stayed for 0.2 s or more was defined as the gaze point in this paper [22,23], and its movement was analyzed. Since pupil diameter varies depending on the person, the ratio of the pupil diameter at the time of watching to the pupil diameter at rest before each subject watched the video was calculated.

#### 2.3.3. Measurement of Autonomic Nervous System

As mentioned above, saliva release is closely related to the autonomic nervous system. Therefore, we measured the activity of the autonomic nervous system from the heart rate obtained using an electrocardiograph. The equipment utilized was a multi-channel telemeter system WEB-1000 by Nihon Kohden Corporation. Regarding this equipment, it is possible to make a measurement by electrocardiography on unconstrained conditions, such as heart-rate signal, which was obtained by using a “Telemeter Picca”, which is a sensor attached to the body surface and transmitted to the processor wirelessly. To analyze the signal, MemCalc/Tarawa, an analysis software by GMS Corporation, was utilized [24]. This software conducts a spectrum analysis of heart-rate signal through fast Fourier transformation and separates the signal into low-frequency components LF: 0.04–0.15 Hz and high-frequency components HF: 0.15–0.4 Hz. Then, the ratio of HF components against two frequency components, such as LF and HF, and the value (LF/HF) of a low-frequency component divided by a high-frequency component are outputted. The value shown as HF is the activity of the parasympathetic nervous system. The value shown by LF is influenced by both the sympathetic nervous system and the parasympathetic nervous system. Based on this, the value shown as LF/HF, which is the ratio between LF and HF, is said to be an activity of sympathetic nerves [25].

#### 2.3.4. Measurement of Brain Activity

The prefrontal area controls thinking, recollection of episodes, decision making, management of emotion, as well as centralized management of conscience or attention [26]. In addition, Sato, et al. clarified that salivary-gland activity on the parotid gland plays a big role in changes in cerebral blood flow, which were measured near the temple, caused by smell stimulation [27]. With reference to these studies, we focused on the prefrontal cortex and the temple area and investigated the differences in activities for each location. The wearable optical topography WOT-100 by NeU Corporation [28] was used to measure oxygenated hemoglobin in the brain, which could be an index of brain activity (Figure 2—S1). The device was used by wrapping it around the forehead so as to conduct a measurement through 16 channels in not only the prefrontal cortex but also even near the temple. Principally, a measurement was conducted based on near infra-red spectroscopy (NIRS). Near infrared light (wavelength: 700–900 nm) was irradiated in the skull to detect the weakened light, which had moved a certain distance in brain, through a light-receiving sensor. By analyzing the light-sensor output, it is possible to convert it into the blood-flow amount. Based on this detected light, the change in oxygenated hemoglobin concentration of the cerebral cortex (Oxy-Hb), deoxygenated hemoglobin concentration (Deoxy-Hb), and total hemoglobin concentration can be measured. However, as it is not possible to measure an optical path length from irradiation to detection, obtained data are not the absolute value of hemoglobin but the relative concentration change. The device has a function that can topographically display the hemoglobin change measured by the sensor in real time [29].

### 2.4. Food Video Content for Evaluation

**Figure 3:**
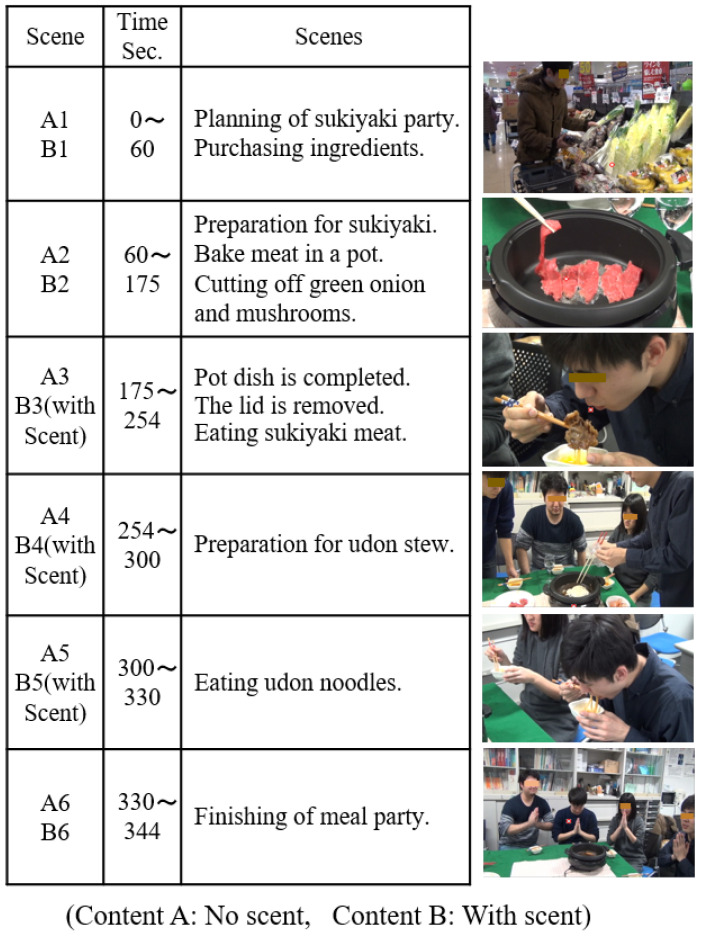
Story lines of sukiyaki gourmet videos.

Figure 3 shows food videos based on “sukiyaki” used in the experiment. The story was created with reference to the popular Japanese gourmet TV program “Solitary Gourmet, Season 5, Episode 12” [30]. According to timely research, “sukiyaki” is the most popular hot pot dish among Japanese people [31]. The video consists of six scenes and runs for five minutes and 44 s in total. In scene 1, performers plan to have a sukiyaki party and buy foodstuffs for sukiyaki at the supermarket. This is a familiar scene everyone experiences at home, and the subject’s appetite is expected to be weak. In scene 2, they grill meat in the pan, adding sukiyaki sauce and cut vegetables. Thus, they start cooking with a lid on the pan. In this scene, the “sukiyaki pan” impresses us so concretely that it is expected to enhance our appetite little by little. In scene 3, when the sukiyaki is ready, the lid is removed. Four performers pick up meat and vegetables with chopsticks one after another for consumption. At this point, it is expected that the subject will imagine a good scent and generate a strong appetite. In scene 4, a ball of udon noodles is put in the soup remaining in the pan after all the foodstuffs of sukiyaki are consumed. As it is a common cooking scene, it is expected rather suppress appetite compared to the meal scene. In scene 5, performers eat udon noodles. This scene is expected to stir up a strong appetite again. Scene 6 includes a greeting after the sukiyaki party. We labeled the video without scent as content A, and that after scene 3, including sukiyaki scent, as content B.

### 2.5. Method of Displaying Scent

When sukiyaki is cooked, usually soy sauce, sweet sake, and sugar are used as seasonings. “Sukiyaki sauce” is a mixed seasoning composed those ingredients that makes it easy to cook sukiyaki. This is why we used the sauce for the display of scent of sukiyaki. The sauce is put in a container, and we heat and vaporize it. The scent of sukiyaki in the container is sucked up by an air pump and sent to the bamboo blind screen of the air-flow control device through the solenoid valve. The odor intensity can be controlled depending on the volume of sauce and the heating temperature. Regarding content B, we opened the solenoid valve at the starting point of scene 3 so that the smell came out of the slit of the screen. As we sent a light breeze at wind speed below one meter from the slit to the viewer, the smell was displayed just in front of the face of the viewer. Regarding the odor intensity, we conducted a preliminary sensory evaluation test so that the subjects could clearly recognize the odor as sukiyaki sauce.

## 3. Experimental Method Using Subjects

**Figure 4:**
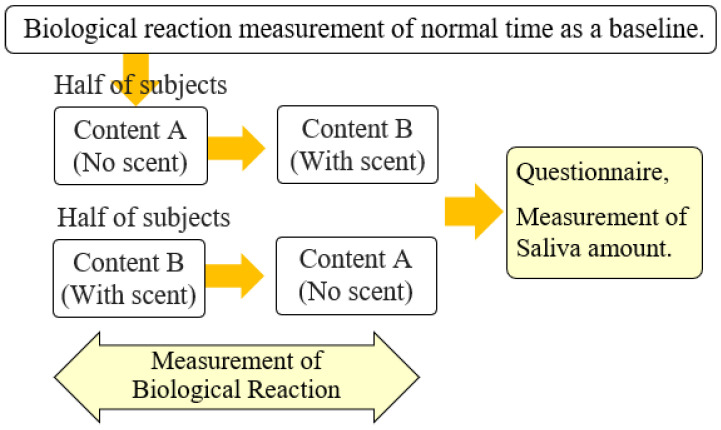
Experimental procedure.

In this study, we thought that the realistic sensation in the favorite food video would correspond to appetite, accompanied by taste and saliva of the subject. Therefore, in this experiment, we focused on how appetite changes while watching a video, whether there is a difference in saliva flow rate, length of video viewing, differences in excitement and relaxation depending on the scene, and whether the scent presentation improves appetite. Our experiment method is shown in Figure 4. Prior to the experiment, a preliminary questionnaire was conducted to confirm the mental state and food preferences of subjects. After checking that the subjects were in good health and did not dislike sukiyaki food, they were made to wear NIRS and an electrocardiograph sensors. Then, for the purpose of checking saliva production at a normal baseline, we had them put saliva absorption sheets in their oral cavity and spend time relaxedly. When the same time period as the video duration had passed, they were asked to take the sheets out of their mouths so that the weight of saliva at a normal baseline could be measured, based on a change of weight before and after absorption.

Then, we had each subject sit in a chair located in front of the screen in the evaluation room. Videos were displayed using the KMMD. The viewing distance was 1.5 m. As mentioned above, the scent was presented to the subject 1.5 to 2 s after the release, as it was placed in an air flow of 1 m or less per second. During the experiment, we kept the surroundings dark and let the subjects focus on the video. Therefore, the biological reaction measuring device was not in the field of view. The abovementioned eye-mark recorder was not attached and did not burden the subject. Additionally, since the electrocardiograph is wireless, the subject did not have to be aware of it while wearing it. Among the measuring means, those that could impose a burden on the subject are a saliva measurement sheets and the NIRS device worn on the head to measure cerebral blood flow. The subjects were given sufficient explanation to relieve anxiety and were encouraged to relax before the experiment. Furthermore, during the experiment, the subjects were instructed to concentrate on the video. Moreover, when they felt a sensation of deliciousness while viewing the video or when they felt saliva secretion, they were ordered to raise their left index finger. Those in charge of the experiment were located in the back of the room, which was invisible to subjects, and checked for the finger movement and monitored the measuring equipment. This made it possible to record the relationship between the subject’s sensation and the reaction time of the device. After checking the mental state of subjects based on the reaction of measuring equipment, we had them hold the saliva absorption sheets in their mouth and watch the video. When the video finished, subjects were ordered to take the sheets out of their mouth. Measurement of saliva secretion was conducted based on change of weight before and after video display. Additionally, for each scene, subjects answered a seven-questionnaire about their appetite. Specifically, they were asked how much appetite, accompanied by taste and saliva, was felt with each scene. In addition, they were asked to freely answer what they were thinking while watching the video, whether they remembered a past experience of sukiyaki pot (episodic memory), and whether there was anything related to the measuring means that impaired their sense of reality.

The subjects were 10 Japanese university students aged 21 to 22 years (5 males and 5 females) who participated in the experiment more than 2 h after the previous meal and before the next meal. Contents A and B differ in the presence or absence of scent, but in order to prevent the displaying order of videos from influencing the result, the subjects were randomly divided into two groups (five subjects per group). One group watched the video in the order of A to B, while the other watched B to A. The experiment data for ten subjects were gathered and statistically processed.

This study is based on an experiment that was conducted with human subjects to evaluate the sense and biological reaction measurement. Therefore, we obtained approval from Tokai University Ethics Committee for “a study geared to humans”, as well as the informed consent of all the subjects prior to carrying out the experiment.

## 4. Experimental Results and Discussion

### 4.1. Results of Questionnaire on Appetite

**Figure 5:**
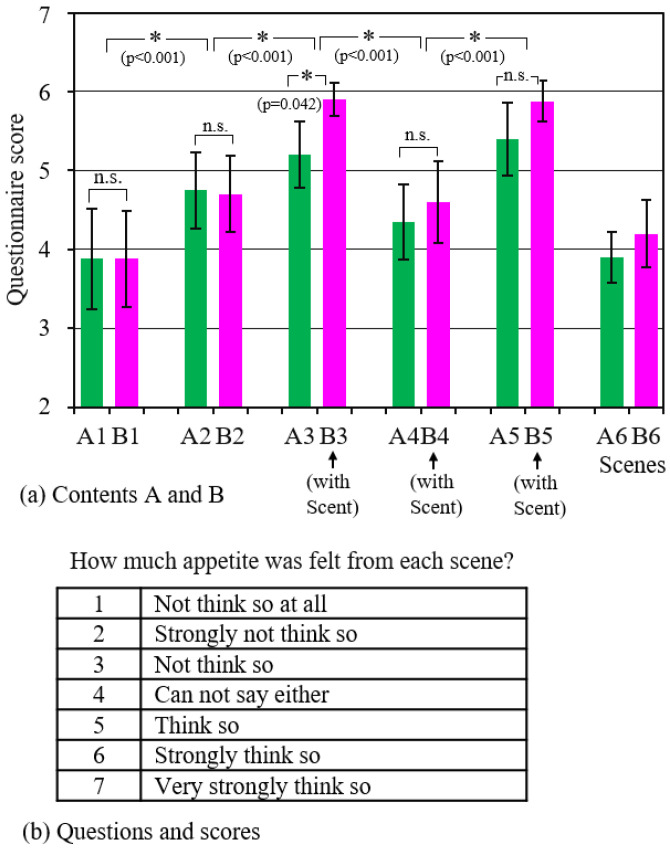
Questionnaire results for each scene.

Figure 5 shows the results of the questionnaire about the abovementioned appetite in each scene immediately after watching videos. Figure 5a shows mean values and standard deviations of the evaluation scores, and Figure 5b shows the questionnaire. A1 to A6 show the six scenes in content A, while B1 to B6 show the scenes in content B during which scent was displayed. Differences in mean values were tested at a significance level of 5% using two-way ANOVA with scene and scent factors. IBM SPSS Statistics was used for the analysis. There were five levels of scenes (scenes 1 to 5) and two levels of scent (with/without). Scene 6 was excluded from the analysis because it is not directly related to cooking and eating. Therefore, it was a test of the difference of 10 groups in total. As a result, the difference between the mean values due to both factors became clear (scene: F(4,90) = 49.5, *p* < 0.001, scent: F(1,90) = 8.52, *p* = 0.004). In addition, one-way ANOVA showed that there were differences in each group (F (9,90) = 23.9, *p* < 0.001). Furthermore, Tukey’s method was used for multiple comparisons. Combinations with clear statistical differences are indicated by *. N.s. is a combination where the difference was not statistically significant because *p* > 0.05.

As described above, it was found that the evaluation score changes depending on the scenes. The score became slightly higher when cooking began (scene_2), with the preparation of ingredients (scene_1), and it became even higher during the eating scenes (scene_3 and scene_5). Regarding the reason why the score of scene_4 is lower than those of scenes_3 and 5, as mentioned in 2.4, it is thought that the impact on the subject was rather small because it was not a meal scene but an udon cooking scene, which is the latter half of the sukiyaki hot pot course. In addition, a comparison of A3 and B3 revealed that the scent presentation increased the evaluation score.

In the free opinion questionnaire, many subjects answered that they perceived the scent and felt the image of “sukiyaki pot” more realistically and recalled the memories of the past. It is considered that the scent acted psychologically to reinforce the reality of the image since the scent was presented at the right time when the pot was boiled. In addition, there was no opinion expressed that it was not possible to concentrate on the video due to the burden of mounting the measuring device.

### 4.2. Change in Saliva Amount

**Figure 6:**
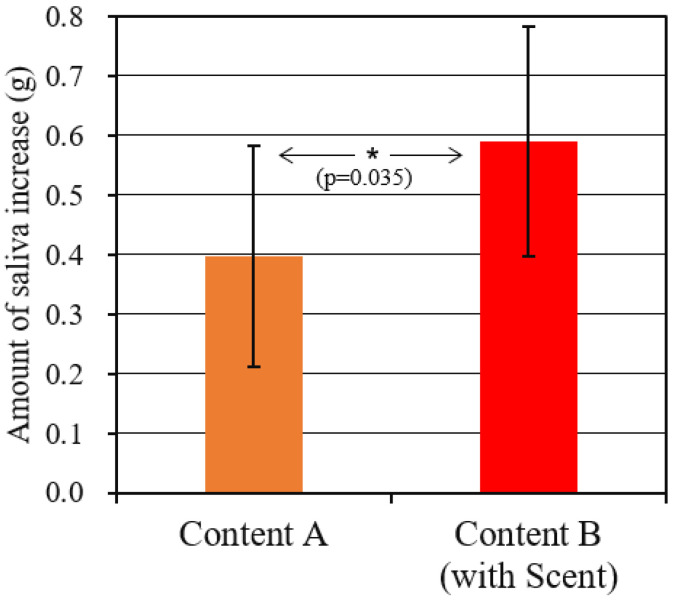
Increase in saliva due to dish viewing.

Figure 6 shows the change by weight in saliva volume during video viewing with respect to the normal saliva volume. The figure shows the mean and standard deviation of all subjects. For both content A and B, a t-test of one sample was performed to clarify whether the change in saliva flow rate was statistically significant. As a result, it was found that the saliva flow rate increased at a significance level of 5% in both of the two conditions (content_A: t(9) = 6.76, *p* < 0.001, content_B: t(9) = 9.65, *p* < 0.001). Furthermore, when comparing contents A with B as shown by * in the figure, it was confirmed by Welch’s *t*-test that the amount of increase was larger in content B, in which the scent was presented, at a significance level of 5%. (t(18.0) = 2.28, *p* = 0.035).

As seen in Figure 5, each content includes some realistic scenes in which appetite was strongly felt. Moreover, in content B, during which the scent was presented, the appetite was higher. As shown in Figure 6, the saliva flow rate actually increased, consistent with this result. In the future, if the saliva flow rate and the components are measured for each scene, a more detailed relationship with appetite may become clear.

### 4.3. Distribution of Gaze Point and Change in Diameter of Pupil

**Figure 7:**
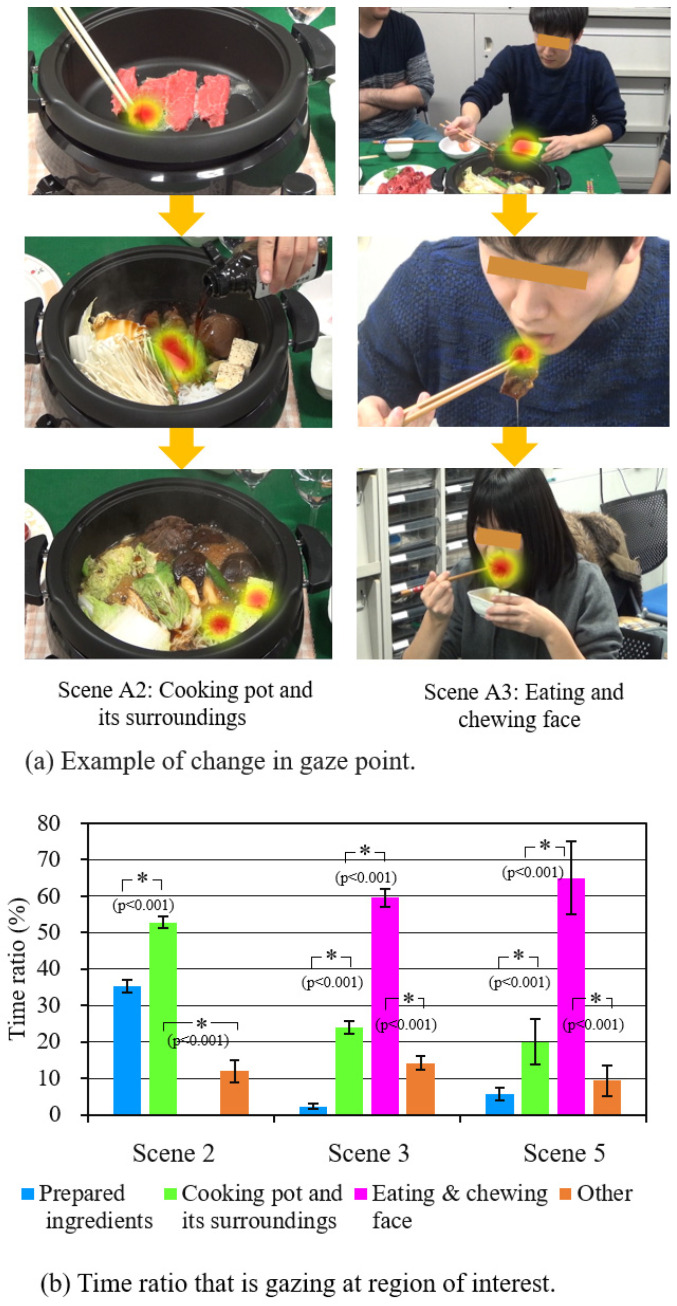
Gaze-point measurement results.

Figure 7 shows the results of measuring gazing points while the subject was watching the content. An example of gaze-point change is shown by a group of red dots in Figure 7a. In scene A2, the subject’s gaze focused on the pot and moved, as if they were looking for their favorite ingredients. In scene A3, the gaze points of the subject followed the action of a performer who picked up a slice of meat with chopsticks, put it in the egg, and chewed it in the mouth, without paying attention to anything else.

Figure 7b shows the mean values of the time ratio where the subject focused on four target areas on the screen in scenes 2, 3, and 5 of content A and the standard deviation. If the gaze-time ratio is large, it is presumed that the area was of great interest. One-way ANOVA with four target areas as levels showed that there was a difference in the mean values (scene_2; F(3,36) = 1464.5, *p* < 0.001, scene_3; F(3,36) = 1865.6, *p* < 0.001, scene_5; F(3,36) = 186.2, *p* < 0.001). As a result of multiple comparisons, the combinations in which a difference was confirmed at a significance level of 5% are indicated by * in the figure. It can be seen that most of the subjects were highly interested in the inside of the pan in scene 2 and the performer’s eating behavior in scenes 3 and 5.

**Figure 8:**
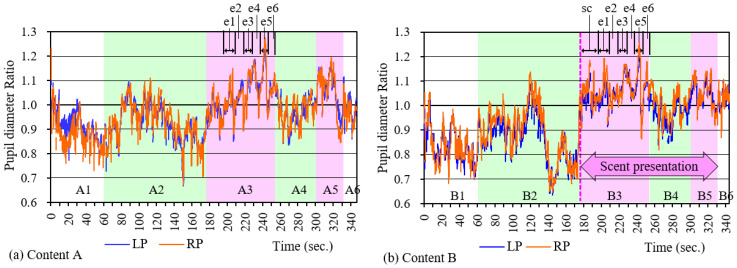
Example of change in pupil diameter during content viewing.

Figure 8a,b show an example of a change in pupil diameter during the time a subject watched contents A and B, respectively. Blue color (LP) shows the pupil diameter of the left eye, while red (RP) shows that of the right eye. There is not a big gap between them, and their movement seems almost the same. In the beginning of scenes A2 and B2, where cooking started and meats were thrown into the pan to be grilled, a temporal mydriasis occurred, and a surge of interest with tension and excitement was considered. Then, after a while, miosis occurred during the part of the scene where the vegetables were cut, put in a pot, placed under a lid and left to boil. In scenes A3 and B3, where the pan lid was taken off and three performers ate one after another (1st performer: e1, e2, 2nd performer: e3, e4, 3rd performer: e5, e6), a large extent of mydriasis was observed. As for B3, which displays scents in conjunction with the opening of a lid, mydriasis was remarkable. “sc” in the figure shows the reaction of starting scent presentation. Looking at B3 in detail, large mydriasis occurred multiple times during scenes e1, e3, and e5, where the meats were put in the three performers’ mouths. Miosis occurred multiple times during scenes e2, e4, and e6, where the actor was chewing. In this way, even during a scene that tends to have mydriasis as a whole, it can be seen that mydriasis and miosis were repeated in a short time, reflecting the video content. At this moment, many subjects, including the above one, gave a finger sign (mentioned above) indicating that they felt saliva secretion. In scene 4, where udon was prepared to cook in the soup, miosis was again observed, while during scene 5, where they ate udon, the abovementioned mydriasis was observed. 

**Figure 9:**
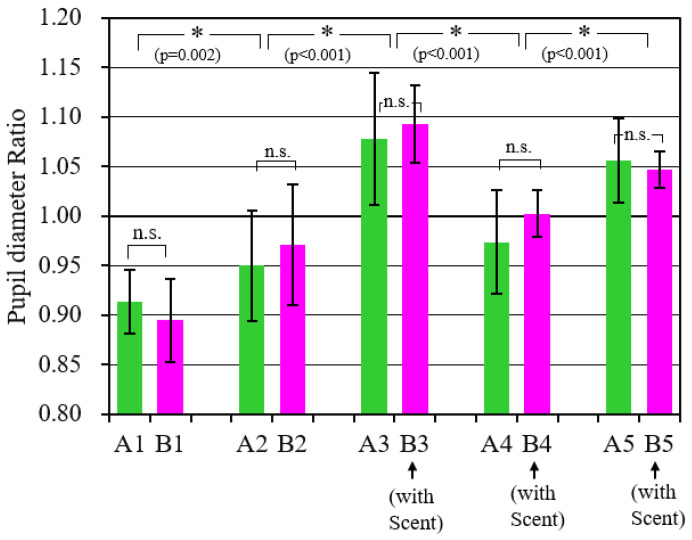
Comparison of pupil diameters during each scene.

Figure 9 shows the mean value and standard deviation of the pupil diameter change ratio of all the subjects from scenes 1 to 5. The difference between these mean values was tested at a significance level of 5% using a two-way ANOVA with scenes and scents as factors. As a result, the difference in the scene (scene; F(4,90) = 49.2, *p* < 0.001) was confirmed, although no statistically significant difference was confirmed in terms of with or without scent (scent; F(4,90) = 0.64, *p* = 0.426). Combinations in which statistically significant differences between scenes were affirmed using the multiple comparison method are indicated by * in the figure. Also, n.s. indicates the combinations where a statistically significant difference could not be confirmed because *p* > 0.05. From this analysis, it was found that the pupil diameter changed to reflect the content of the scene. In the cooking scenes (A2, B2, A4), the rate of change in pupil diameter was 1.0 or less, so the pupil tended to be miotic. During eating scenes (A3, B3, A5, B5), mydriasis tended to occur, as it was 1.0 or higher. It seems that excitement and tension temporarily occurred during scenes 2 to 3 and scenes 4 to 5.

### 4.4. Change in Autonomic Nervous System

**Figure 10:**
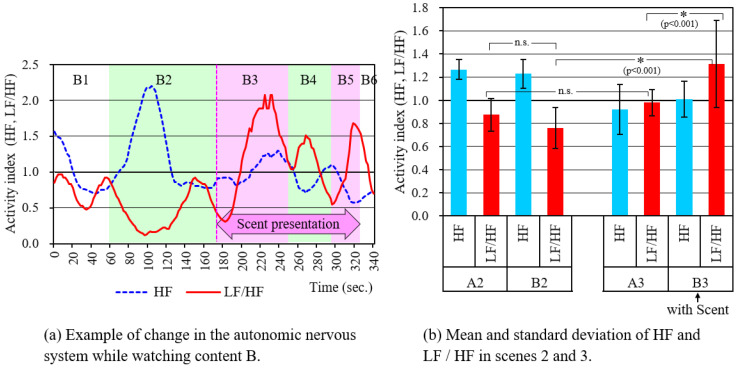
Autonomic nervous system measurement results.

Figure 10 shows the measurement results of the autonomic nervous system. Figure 10a is an example of changes in the activity index of the parasympathetic nervous system and the sympathetic nervous system (HF and LF/HF, respectively) when a subject was watching content B. Since there are large differences between subjects regarding the responses of HF and LF/HF, the values normalized by the mean value during the experiment for each subject were used as the activity index. Therefore, they are the relative values for each scene. It was found that in scene B2, the parasympathetic nervous system was enhanced and the sympathetic nervous system was suppressed, while in scenes B3 and B5, the sympathetic nervous system was enhanced and the parasympathetic nervous system was suppressed. Changes in LF/HF, which is an index of sympathetic nervous system activity, are similar to changes in pupil diameter (Figure 8b). The reason seems to be that the dilator muscle of the pupil is controlled by the sympathetic nervous system.

Figure 10b shows the mean value and standard deviation of the activity indexes when each subject was viewing scenes 2 and 3. The difference in mean values was tested by analysis of variance with a significance level of 5%, using scenes, scents, and autonomic nervous activity index as factors. With the results, it became clear that there are differences due to the following factors: activity index (F (1,72) = 8.42, *p* = 0.005), scene x activity index (F (1,72) = 49.8, *p* < 0.001), scene x scent (F (1,72) = 11.6, *p* = 0.001). Focusing on LF/HF, the combinations for which statistically significant differences were confirmed as a result of multiple comparisons are indicated by * in the figure. N.s. is a combination where the difference was not statistically significant because *p* > 0.05. The activity index of the sympathetic nervous system was greater in the eating scene, B3, when the scent was presented than in the cooking scene, B2. At this time, tension and excitement may have occurred, which is consistent with the results of the questionnaire and the results of the pupil-diameter measurement.

Comparing the estimate of change in the autonomic nervous system by using the pupil diameter data from the eye camera and by using electrocardiograph data, while the eye camera directly measures the pupil diameter, the electrocardiograph needs to process the fluctuation of the R-R interval [24], so the response for estimation seems to be faster in the former [32]. In addition, as it enables us to consider the relation to the gaze target, an eye camera is more appropriate when evaluating videos displayed in this experiment. However, it has been pointed out that the method using pupil diameter is problematic for estimation accuracy of the sympathetic nervous system activity. Thus, further study is required [33].

### 4.5. Activation of Prefrontal Cortex

**Figure 11:**
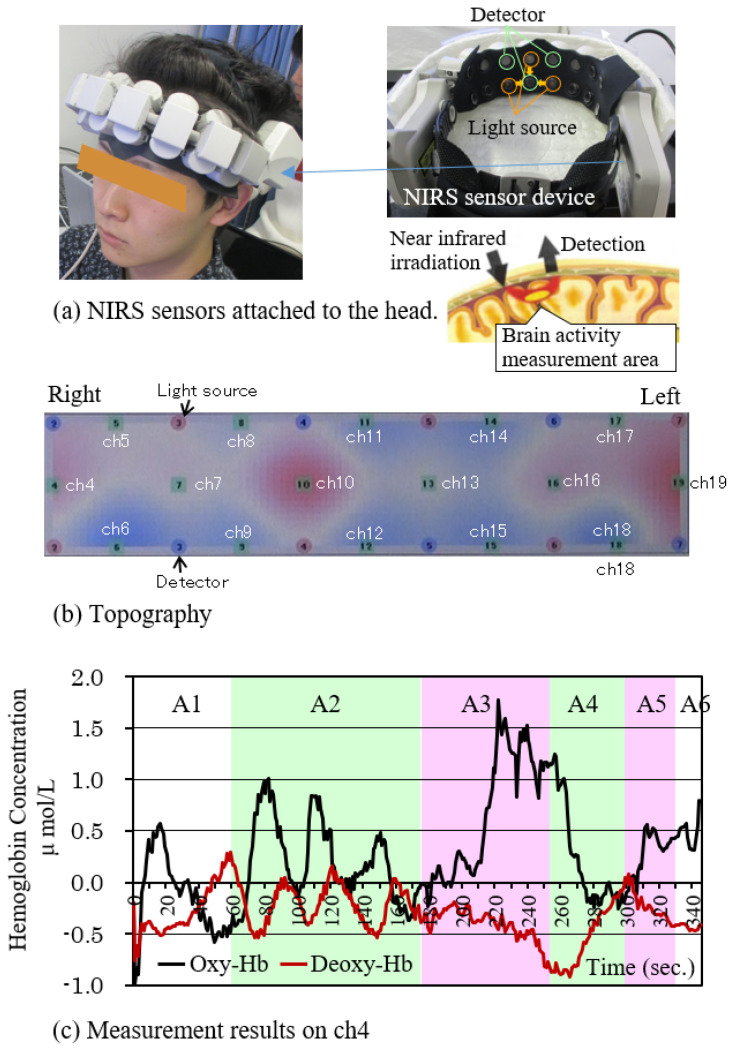
Example of multichannel NIRS results while viewing the content.

Figure 11 shows the measurement results of optical topography WOT-100. Figure 11a shows the measurement principle of the NIRS device, the arrangement of the multi-sensors, and the state of being set on the head. The measurement range was from the prefrontal cortex to the temple area. Figure 11b shows a topography of oxygenated hemoglobin concentration. Numbers in red circles show the light sources, while those in blue circles show the detectors, and those in green circles show the channels. There are 16 channels in all, the activity of which is indicated by color change. Red indicates the part with high activity. The figure is an example of the result of the moment when the performer ate meat in scene 3 of content A. Reactions were seen on ch10, which corresponds to the dorsolateral part of the prefrontal cortex, and ch4 and ch19 near the temples.

Figure 11c is an example of temporal changes of the oxygenated hemoglobin concentration (Oxy-Hb) and the deoxidized hemoglobin concentration (Deoxy-Hb) of ch4. Oxy-Hb was observed to increase during scene A2, where performers grilled meat, and in scene A3, where they ate it. It was especially remarked on in scene A3. It showed a similar process to pupil reaction, as described in Figure 8a, which means that subjects’ interest in images increased during scenes 2, 3, and 5. A similar trend was obtained for content B. The prefrontal cortex dorsolateral part is said to play an important part in memory, recognition, motivation, and judgment [34]. In addition, it is known that the area around the temples is related to taste and saliva secretion [8,27]. With reference to these facts, it is possible to think that the rise of Oxy-Hb in scene 3 was derived from the fact that subjects seeing a performer eat sukiyaki reminded them of their own eating episodes, inducing their appetite for food. As mentioned above, many subjects gave similar answers in the questionnaire. However, further study is needed to clarify the relationship between episodic memory and brain activity.

**Figure 12:**
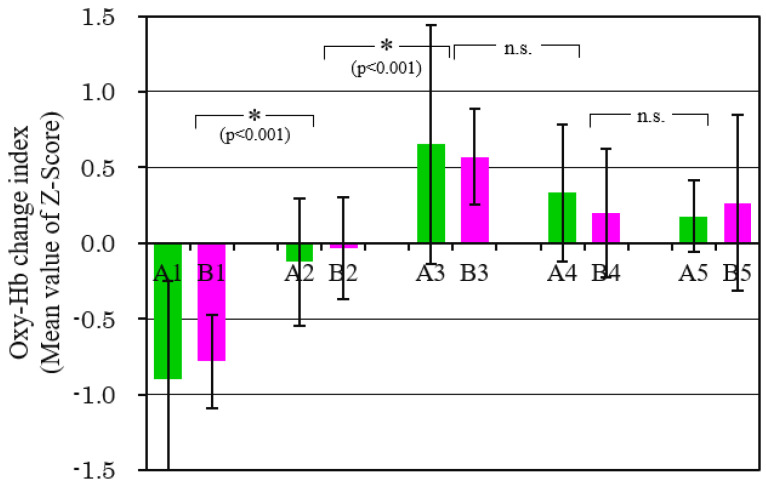
Comparison of Oxy-Hb change index in each scene. (Data of channels 4, 10, 13, and 19 was used).

Figure 12 shows the mean values of the Oxy-Hb change indexes through ch4, ch10, ch13, and ch19 for all the subjects while watching each scene. As mentioned above, ch4 and ch19 are near the temples. ch10 and ch13 are examples of the prefrontal cortex. The vertical axis is the dimensionless standard score (Z-score) [35]. As there is a big difference between subjects regarding Oxy-Hb, the data standardized by the mean value during the experiment were used for each subject. In addition, the sum of standardized Oxy-Hb obtained from the four channels was used as the index of the *y*-axis. Therefore, it shows the relative frontal-lobe brain activity per scene. A two-way ANOVA with scenes and scents as factors was used to test the difference in mean values. As a result, the difference between scent conditions was not statistically significant, but the difference between scenes was clear at a significance level of 5% (F(4,90) = 25.87, *p* < 0.001). As a result of multiple comparison using Tukey’s method, the combinations in which the difference became clear at a significance level of 5% are indicated by *. N.s. is a combination where the difference was not statistically significant because *p* > 0.05. The index increased in scene 3 as compared with scene 2. It can be thought that in the scenes including eating behavior, the brain should be easily activated due to empathy compared to the scenes of only cooking.

### 4.6. Correlation of Each Measurement Method

To objectively evaluate the presence of the impression of “being there” while watching food videos, we calculated the correlation between the questionnaire results, which are subjective evaluations, and the biological reaction measurement results. Table 1 shows Pearson’s correlation coefficient. 

Each correlation coefficient is significant at the 1% level. The results of pupil diameter and NIRS showed a moderate or higher correlation with the questionnaire results. In addition, the sympathetic nerve index LF/HF showed some correlation. These analysis results suggest the possibility of objectively evaluating the presence of food videos.

## 5. Conclusions

We presented food videos to the subjects using KMMD, examined a method of objectively evaluating the realistic sensation by appetite accompanied by saliva, and clarified the following.

(1) Appetite was greatly influenced by the scene; the evaluation score increased during the eating scene. Moreover, when the scent was presented during the scene, the score further increased.
(2) An increase in saliva flow rate was associated with watching videos. It increased even further when scents were displayed.
(3) The subject’s gaze points were concentrated as if the performer’s eating behavior was traced. Pupil diameter was greatly influenced by the scene; mydriasis and miosis were repeated. During the eating scene, there was a tendency for mydriasis to occur temporarily.
(4) In the measurement of the autonomic nervous system, the parasympathetic nervous system tended to be enhanced during the food-preparation and cooking scenes, and the sympathetic nervous system tended to be enhanced during the eating scene. This tendency was promoted by the presentation of scents. The activation of the parasympathetic nervous system is presumed by the subject’s relaxed state. They may be enjoying the cooking process. In addition, the tension and excitement of the subject are presumed during the scene where the sympathetic nerve is enhanced.
(5) As shown by optical topography, oxygenated hemoglobin concentration in the prefrontal cortex and near the temples increased during the scene where appetite increased, which shows brain activity acceleration. It can be considered that a temporal tension or an excitement occurred due to empathy for the performers. The emotional transfer to the performers may have occurred, and they may have been excited to imagine their own meals.
(6) It was clarified that a moderate or higher correlation occurred between the questionnaire results regarding appetite and the biological reaction measurement results.

As mentioned above, KMMD properly displays with the scent as if there were a delicious sukiyaki pot there. Furthermore, the realistic sensation regarding favorite food videos might be estimated from biological reaction measurements. In particular, the line-of-sight measuring device (eye camera) is convenient for easily estimating psychological changes, as it can measure the gaze point and the pupil diameter at the same time without having to be worn by subjects. Since KMMD has small slits or holes on the screen, in the future, by installing an eye camera here, psychological measurement and presentation of images and scents will be possible in real time on one display device [36]. If the psychological state estimation result is fed back to the presented content, it can be expected to be applied to interactive advertisements where there is communication with customers. For example, a service in which the display side estimates the appetite of the person viewing the advertisement and introduces the store can be considered. In the future, we would like to increase the types of subjects and videos to improve reliability.

## Figures and Tables

**Table 1:** Correlation coefficient between each measurement method.

	Question-Naire	Pupil Diameter	LF/HF	NIRS
Questionnaire	1.0	0.720 (*p* < 0.001)	0.563 (*p* = 0.01)	0.630 (*p* < 0.001)
Pupil diameter		1.0	0.576 (*p* = 0.008)	0.659 (*p* < 0.001)
LF/HF			1.0	0.584 (*p* = 0.007)
NIRS				1.0
